# The Clinical Characteristics and Prognosis of AYA and Older Adult ETP-ALL/LBL: A Real-World Multicenter Study in China

**DOI:** 10.3389/fonc.2022.846573

**Published:** 2022-06-06

**Authors:** Jinyan Xiao, Zihong Cai, Hao Wang, Xuekai Li, Biqi Zhou, Yujie Liu, Ying Wang, Peipei Xu, Li Wang, Depei Wu, Liping Dou, Hongsheng Zhou, Yang Xu

**Affiliations:** ^1^ Jiangsu Institute of Hematology, National Clinical Research Center for Hematologic Diseases, The First Affiliated Hospital of Soochow University, Collaborative Innovation Center of Hematology, Suzhou, China; ^2^ Department of Hematology, Nanfang Hospital, Southern Medical University, Guangzhou, China; ^3^ Department of Hematology, Chinese People’s Liberation Army (PLA) General Hospital, Beijing, China; ^4^ Department of Hematology, Nanjing Drum Tower Hospital, Clinical College of Nanjing Medical University, Nanjing, China; ^5^ The Youth Committee of the Chinese Society of Hematology, Suzhou, China; ^6^ Shanghai Institute of Hematology, State Key Laboratory of Medical Genomics, National Research Center for Translational Medicine at Shanghai, Ruijin Hospital Affiliated to Shanghai Jiao Tong University School of Medicine, Shanghai, China

**Keywords:** early T-cell precursor, leukemia, multicenter, induction, allo-SCT

## Abstract

Early T-cell precursor (ETP) lymphoblastic leukemia/lymphoma is a high-risk T lymphoblastic leukemia/lymphoma (T-ALL/LBL) subgroup. We performed a real-world multicenter study to explore the clinical characteristics and prognosis of adolescent and young adults (AYA) and older adult ETP leukemia/lymphoma. A total of 103 patients with ETP-ALL/LBL in five centers in China between January 2016 and February 2021 were included in this study. The median age was 29 years (range, 15–70 years). Next-generation sequencing was performed in 94 patients and revealed that NOTCH1 (35.1%, 33 cases) was the most frequently mutated gene, followed by JAK3 (16.0%, 15 cases), PHF6 (13.80%, 13 cases) and EZH2 (11.70%, 11 cases). Complete remission (CR) was obtained in 74.2% (72/97) of patients, and 6 relapsed/refractory patients received a decitabine combined with AAG priming regimen as reinduction therapy with a CR rate of 50%. With a median follow-up of 18 months (0.5–60 months), the 2-year overall survival (OS) and relapse-free survival (RFS) rates for the entire cohort were 54% and 57.7%, respectively. Allogeneic stem cell transplantation (allo-SCT) was performed in 59.8% (58/97) of patients. After landmark analysis at 6 months, the 2-year OS rates was 77% of patients with allo-SCT at CR1 and 25% of patients with chemotherapy alone (p < 0.001). A multivariate analysis suggested that allo-SCT and CR after the first course induction were independent prognostic factors for OS. Collectively, we reported the largest cohort study with AYA and older adult ETP-ALL/LBL, and we found that ETP-ALL/LBL was highly invasive and had a poor long-term prognosis. Allo-SCT could significantly improve ETP-ALL/LBL patient survival.

## Introduction

Early T-cell precursor lymphoblastic leukemia/lymphoma (ETP-ALL/LBL) is a hematological malignancy originating from immature T lymphoblastic cells. ETP-ALL has been reported in 13~16% of T cell acute lymphoblastic leukemia (T-ALL) cases in children (1, 2) and 17~32% in adults (3, 4). In China, ETP-ALL accounts for approximately 45% of adult T-ALL (5). ETP-ALL/LBL has a unique gene mutation expression profile, mainly manifested as a higher incidence of mutations in genes regulating cytokine receptor and RAS signaling, which was similar to the mutational spectrum of myeloid tumors (6). Clinically, ETP-ALL/LBL is highly invasive and has a poor long-term prognosis. St. Jude Children’s Hospital reported that the 10-year remission failure or hematological relapse of children with ETP-ALL was higher than that of children with typical T-ALL (72% vs. 10%) (1). Similar results were found in other studies (3, 7). However, recently, some studies have found that the prognosis of ETP-ALL was not significantly different from that of typical T-ALL (5, 8, 9). The largest published report on ETP was from Zhang et al., in which 53 ETP-ALL patients were included. The 2-year overall survival (OS) was 40.7% ± 8.2% in ETP and 37.9% ± 7% in non-ETP (P>0.05) (5).

At present, ETP-ALL/LBL is still regarded as an important problem with poor long outcome, and there is no standard regimen in this setting, especially for relapsed/refractory ETP-ALL/LBL. Here, we present the clinical characteristics of a multicenter analysis of 103 ETP-ALL/LBL patients to identify potentially useful prognostic factors, assess allogeneic transplantation benefits and explore possible effective treatment options for R/R ETP-ALL/LBL.

## Patients and Methods

### Patients

**Figure 1:**
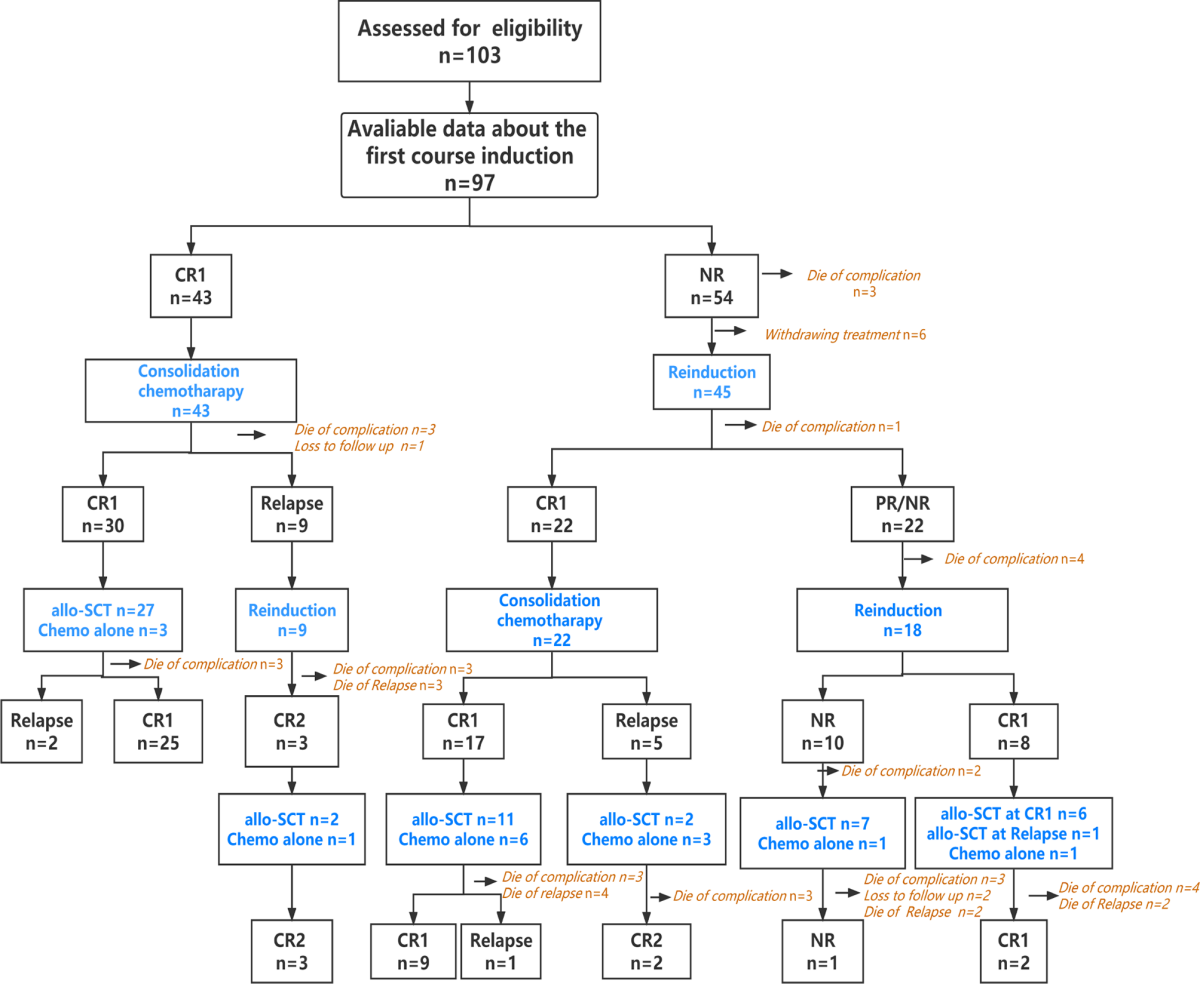
Flow chart of ETP in this study.

Data were collected from five institutions: The First Affiliated Hospital of Soochow University, Nanfang Hospital of Southern Medical University, General Hospital of PLA, Nanjing Drum Tower Hospital and Ruijin Hospital Affiliated to Shanghai Jiao Tong University School of Medicine. During January 2016 and February 2021, 103 *de novo* ETP-ALL/LBL patients 15 years of age or older were included in this retrospective study (**Figure 1**). The diagnosis of ETP-ALL/LBL was based on the 2016 WHO criterion (10). A cutoff of < 20% BM blasts was used to define LBL. The median follow-up time was 18 months (0.5–60 months). The last follow-up time was April 2021. This study was approved by the institutional review board at each institution.

### Immunophenotyping

For patients with ETP, flow cytometry immunophenotyping (FCI) defined the absence of CD1a/CD8, weak expression of CD5 (<75% positive lymphoblasts), and the presence of one or more myeloid or stem cell markers (CD117, CD34, HLA-DR, CD13, CD33, CD11b, or CD65) on at least 25% of lymphoblasts. Minor residual disease (MRD) analysis was performed using flow cytometry detection. We defined 0.01% (1*10^-4^) as the threshold to distinguish MRD-positive from MRD-negative patients: MRD≥0.01% (1*10^-4^) of nucleated cells (NC) was defined as positive, and MRD<0.01% (1*10^-4^) of nucleated cells (NC) was defined as negative (11).

### Next-Generation Sequencing

Genomic DNA was extracted from BM-derived mononuclear cells at diagnosis. The Ion S5 system (Personal Genome Machine, Thermo Fisher, Grand Island, NY, USA) was used to evaluate the panel of 51 common variant gene targets in hematologic malignancies, including ASXL1, ASXL2, BCOR, BCORL1, BIRC3, BRAF, CALR, CBL, CEBPA, C-KIT, CSF3R,CSMD1, DNMT3A, ETNK1, ETV6, EZH2, FBXW7, FLT3,GATA2, IDH1, IDH2, IL7R, JAK1, JAK2, JAK3, KRAS, MPL,MYD88, NOTCH1, NPM1, NRAS, PAX5, PDGFRA, PDGFRB,PHF6, PI6, PIGA, PTEN, PTPN11, RUNX1, SETBP1, SETD2,SF3B1, SH2B3, SRSF2, STAG2, TET2, TP53, U2AF1, WT1 and ZRSR2.The coverage rate of the NGS expander was 98.03%, the depth of the average sequence was 2 500×, and the sequencing depth of more than 95% target regions was 2 000×.

### Treatment

The choice of the treatment regimen was determined by the individual physician based upon CALLG2008 protocol (a protocol developed by the Chinese Acute Lymphoblastic Leukemia Cooperative Group for ALL) and augmented MDACC Hyper-CVAD. Ninety-seven patients accepted induction therapy, including a VDCP-based regime (vindesine 4 mg/d, intravenous infusion, D1, D8, D15, D22, daunorubicin 40 mg/(m2/d), intravenous infusion, D1, D8, D15, D22, cyclophosphamide 1 mg/(m2/d), intravenous infusion, D1, D15, prednisone 1 mg/(m2/d), oral, D1–14, D15–28 (2/3 dose) with or without L-asparaginase 6000IU/m2/d, intramuscular, D11, D14, D17, D20, D23, D26), augmented MDACC hyper-CVAD A protocol (cyclophosphamide, 300 mg/(m2/q12 h), intravenous infusion, D1–3, vindesine, 3mg/m2/d, intravenous infusion, D4, D11, doxorubicin 50 mg/m2, intravenous infusion, D4, dexamethasone 40 mg/d, intravenous infusion, D1–4, D11–14), VDCP-based regime combined with chidamide (chidamide 30 mg, oral, twice per week, vindesine 4 mg/d, intravenous infusion, D1, D8, D15, D22, daunorubicin 40 mg/(m2/d), intravenous infusion, D1, D8, D15, D22, cyclophosphamide 1 mg/(m2/d), intravenous infusion, D1, D15, Prednisone 1 mg/(m2/d), oral, D1–14, D15–28 (2/3 dose) with or without L-asparaginase 6000IU/m2/d, intramuscular, D11, D14, D17, D20, D23, D26) and a DAC combined with AAG priming regimen [decitabine 20mg/m2/d, intravenous infusion, d1-d3, aclarubicin 10mg/d, intravenous infusion, d1-d8, cytarabine10mg/m2/q12h, subcutaneous injection, d1-d14, granulocyte colony-stimulating factor (G-CSF), 50-300μg/d, subcutaneous injection, (when WBC counts are less than 20×10^9/L)]. Patients who were unfit for intensive chemotherapy received dose-reduced chemotherapy regimens. CNS prophylaxis proceeded as needed.

Patients who experienced primary refractory disease or relapse received a reinduction regimen (VDCP-based regimen, augmented MDACC protocol, or a DAC combined with AAG priming regimen (decitabine 20mg/m2/d, intravenous infusion, d1-d3, aclarubicin 10mg/d, intravenous infusion, d1-d8, cytarabine10mg/m2/q12h, subcutaneous injection, d1-d14, granulocyte colony-stimulating factor (G-CSF), 50-300μg/d, subcutaneous injection, (when WBC counts are less than 20×10^9/L)).

After achieving CR, consolidation therapy was carried out, including based on high-dose methotrexate, high-dose cytarabine, L-asparaginase or pegaspargase, and cyclophosphamide. The choice of different consolidation treatment schemes was based on the choice of each center. Allo-SCT was performed in patients with disease status and potential donors.

Among the patients who chose allo-SCT, all received a myeloablative conditioning regimen. Hematopoietic stem cell transplantation included HLA-matched donors and HLA-mismatched donors. A BU/CY-based conditioning regime was as follows: Ara-c 2 g/m2/12h on days -9 to -8, Bu 0.8 mg/kg/6h from day -7 to day -5, cyclophosphamide 1.8 g/m2/d from day -4 to -3, and semustine 250 mg/m2/d on day -10. The TBI/Cy regime consisted of TBI (12 Gy on days -8 to -6), cyclophosphamide 1.8g/m2/d from day -4 to approximately day -3, and semustine 250mg/m2/d on day -9. GVHD prophylaxis consisted of continuous cyclosporine infusion at 3 mg/kg/day starting on day −10, mycophenolate mofetil 1.0 g/day from days −10 to +30, short-term methotrexate administered on days +1, +3, +6, and +11 at dosages of 15, 10, 10, and 10 mg/m2, respectively, and rabbit antithymocyte globulin (ATG) 2.5 mg/kg/d (on days −5 to −2) was administered in the human leukocyte antigen (HLA)-haploidentical related donor (haplo-RD) and unrelated donor groups. The detailed procedure of allo-SCT was reported previously (12, 13).

### End Points

The measured outcomes were complete remission (CR), partial response (PR), no response (NR), overall survival (OS) and relapse-free survival (RFS). CR was defined as <5% bone marrow blasts, no blasts in blood, absolute neutrophil counts (ANCs) >10^9^/L, platelets >10^9^/L and no extramedullary leukemia. PR was defined as bone marrow blast counts >5% but ≤20% and/or persistent evidence of extramedullary disease and a reduction of total lymphoma volume of >50%, no evidence of progression and no new lesions for >3 weeks. NR was defined as bone marrow blast counts >20%, blasts in peripheral blood, failure of hematological recovery, or persistent evidence of extramedullary disease not meeting PR criteria. OS was measured as the time from the date of diagnosis to the date of death or last follow-up. RFS was defined as the time from first CR to relapse, censoring at death in CR or last follow-up. When only allo-HSCT patients were analyzed, the outcome was calculated from the date of transplantation. Refractory disease was defined as refractory to primary therapy or to salvage with intensive combination chemotherapy, first relapse with first remission duration < 12 months, second or greater relapse, or relapse at any time after allo-SCT (14). Relapsed disease was defined as reappearance of blasts in the blood or bone marrow (>5%) or in any extramedullary site after CR. Acute GVHD was graded on the Glucksberg scale from grade I-IV.

### Statistical Analysis

Continuous variables were analyzed by the Mann–Whitney U-test, and categorical parameters were analyzed by Pearson chi-squared test or Fisher’s exact test. OS and RFS were estimated by the Kaplan–Meier method and log-rank test. A landmark analysis for survival was performed to mitigate the effect of early death prior to allo-SCT (15). The landmark time was 6 months (median time from first chemotherapy to allo-SCT). The cumulative incidence of GVHD was estimated with competing risk methods. Relapse was considered a competing risk for GVHD. A P value < 0.05 was considered statistically significant. All statistical analyses were performed using SPSS Version 26 and R Version 4.4.1.

## Results

### Clinical Characteristics

The characteristics of all 103 patients are shown in **Table 1**. There were 73 males and 30 females, and the median age was 29 years (range, 15–70 years). The numbers of patients with ETP-ALL and ETP-LBL were 98 and 5, respectively. The median follow-up time was 18 months (range 0.5–60 months). The median WBC count was 9.09 (0.22–643.0) ×10^9^/L, the median PLT count was 109 (10–413) ×10^9^/L, and the median HGB level was 103.5 (41–172) g/L. The WBC count of 14 patients was ≥100×109/L at initial diagnosis. The LDH of 41 patients was ≥245 U/L. Among the 103 patients, 45 cases (43.7%) had splenomegaly, 49 cases (47.6%) had peripheral lymph node enlargement, and 24 cases (23.3%) had mediastinal masses. The median percentage of bone marrow primitive cells was 80% (4.5% ~ 99%). The karyotypes of 17 patients were complex. 79 patients with treatment were younger than 39 years, they tended to have more splenomegaly and mediastinal masses in clinical manifestations than older adult (**Supplementary Table 1**).

**Table 1:** Baseline characteristics of early T-cell precursor acute lymphoblastic leukemia/lymphoma (ETP-ALL/LBL).

Patient characteristics (n=103)		
Characteristic	Median (ranges)	N (%)
Institution		
The First Affiliated Hospital of Soochow University		45 (43.7)
Nan fang Hospital of Southern Medical University		32 (31.1)
Ruijin Hospital Affiliated to Shanghai Jiao Tong University School of Medicine		3 (3.0)
General Hospital of PLA		20 (19.4)
Nanjing Drum Tower Hospital		3 (2.9)
ETP-ALL		98 (95.1)
ETP-LBL		5 (4.9)
Age	29 (15-70)	
≤39		82 (79.6)
>39		21 (20.4)
Sex		
Male		73 (70.9)
Female		30 (29.1)
WBC × 10^9^/L	9.09 (0.22-643)	
≥100*10^9^/L		14 (13.6)
<100*10^9^/L		88 (85.4)
Unknown		1 (1.0)
Hemoglobin	103.5 (41-172)	
<100 g/l		44 (42.7)
≥100 g/l		56 (54.4)
Unknown		3 (2.9)
Platelet	109 (10-413)	
<35× 109/L		17 (16.5)
≥35× 109/L		84 (81.6)
Unknown		2 (1.9)
LDH, U/L	300.5 (80.2-8082)	
≥245		41 (39.8)
<245		57 (55.3)
Unknown		5 (4.9)
BM blasts (%)	80 (4.5-99)	
≥60		69 (67.0)
<60		29 (28.2)
Unknown		5 (4.9)
Karyotype*		
Normal karyotype		58 (56.3)
Complex karyotype		17 (16.5)
Others		18 (17.5)
Unknown		10 (9.7)
Mediastinal mass		
Yes		24 (23.3)
No		79 (76.7)
Spleen enlargement		
Yes		45 (43.7)
No		58 (56.3)
Peripheral lymph node enlargement		
Yes		49 (47.6)
No		54 (52.4)

WBC, white blood cell; RBC, red blood cell; LDH, lactate dehydrogenase; BM, bone marrow. Karyotype * complex karyotype ≥5 anomalies.

**Figure 2:**
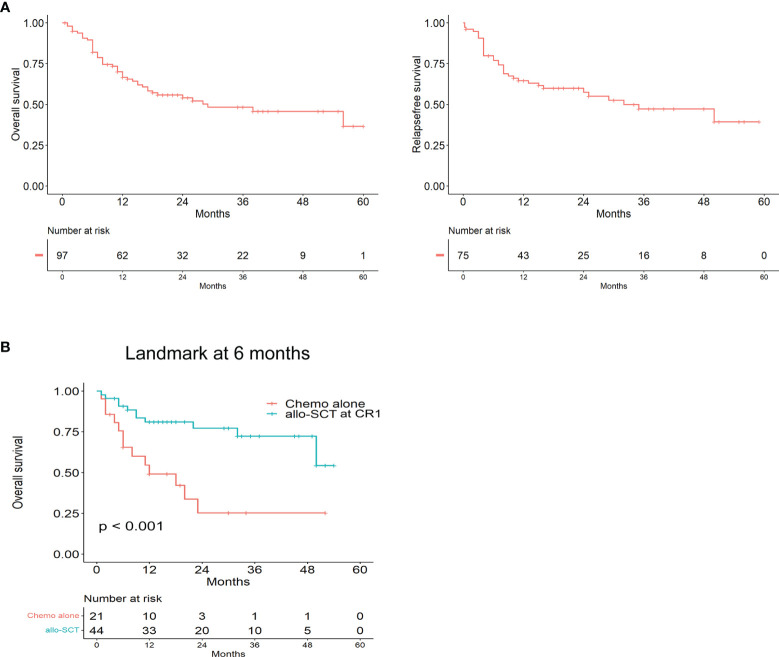
The prognosis of Early T-cell precursor leukemia/lymphoma. **(A)** Kaplan–Meier analyses for Overall survival and Relapse free survival in the whole cohort. **(B)** Overall survival for allo-SCT at CR1 and Chemo alone by landmark analysis, with landmark time at 6 months.

After a median follow-up of 18 months (range, 0.5–60 months), the 2-year OS and RFS rates were 54% and 57.7%, respectively (**Figure 2A**). Patients who presented with ETP-ALL had a median survival time of 38 months compared with 28 months for ETP-LBL. However, this was not statistically significant (P=0.357). The 2-year OS of patients was 77% of allo-SCT at CR1 and 25% of chemotherapy alone (p < 0.001, HR: 0.2833, 95%CI 0.1126 to 0.7130) (**Figure 2B**).

### Gene Mutation Spectrum

**Figure 3:**
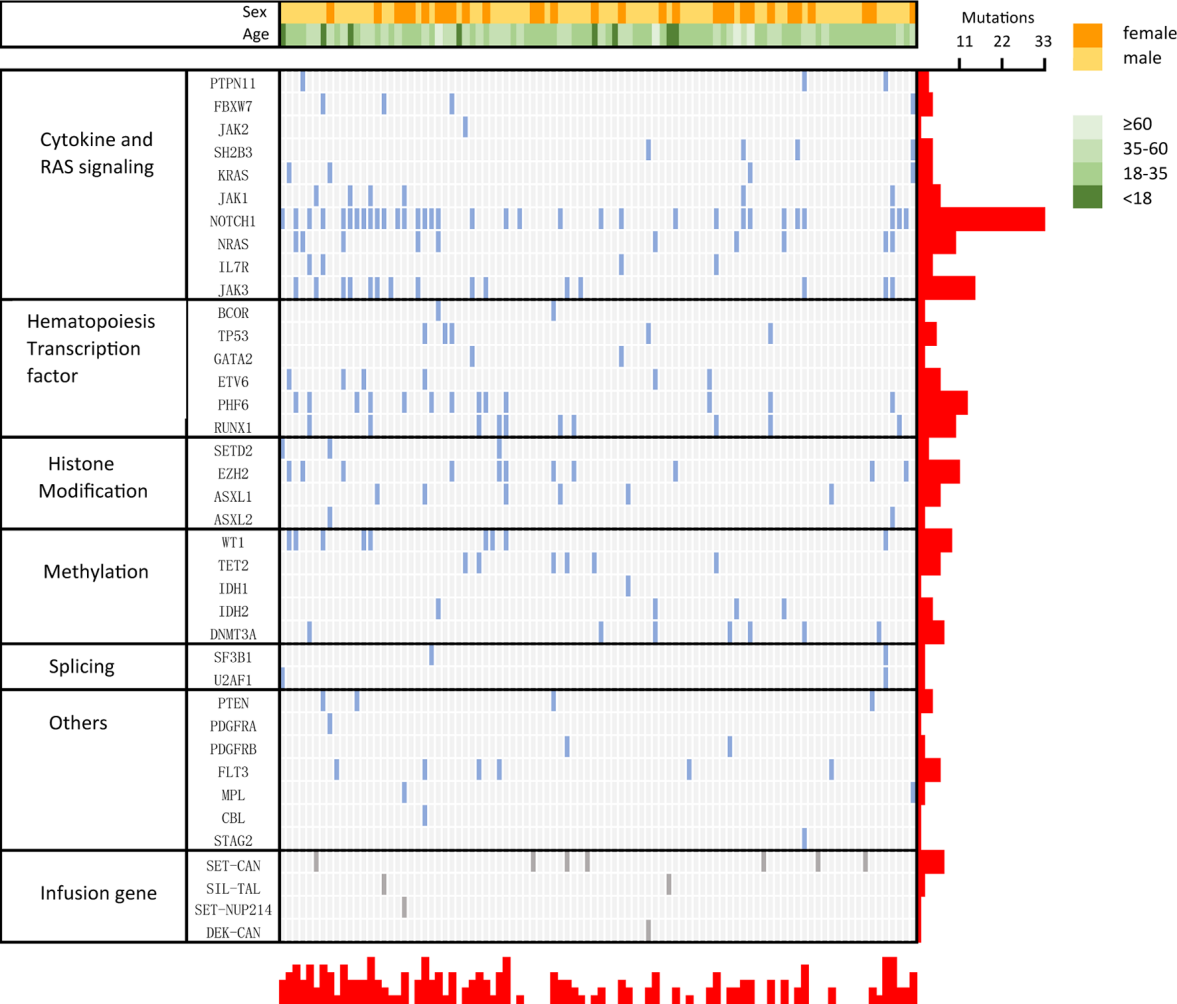
Genetic profile of early T-cell precursor acute lymphoblastic leukemia/lymphoma (ETP-ALL/LBL). Distribution of acquired mutations in 94 patients of ETP-ALL/LBL.

Next-generation sequencing was performed on 94 patients to detect 51 gene mutations, and the results are shown in **Figure 3**, with a median of 2 (0–6) gene mutations. NOTCH1 (35.1%, 33 cases) was the most frequently mutated gene, followed by JAK3 (16.0%, 15 cases), PHF6 (13.80%, 13 cases) and EZH2 (11.70%, 11 cases). Thirty patients did not have gene mutations. Mutated genes were grouped into several signaling pathway. The mutations involved in regulating cytokine and RAS signaling (51.06%), hematopoietic development (30.90%), methylation (26.6%), and histone modification (20%) were found frequently. There was no statistically significant difference in OS between NOTCH1 mutation and NOTCH1 wild-type patients (P=0.299, HR:1.386, 95%CI 0.7151 to 2.686). The most common fusion gene was SET-CAN (7/94). Six out of 7 patients with SET-CAN in our cohort achieved CR after the first induction treatment, and 5 of them successfully experienced allo-SCT in CR1. During the follow-up, 4 patients were still in relapse-free survival. Two patients died of relapse and aGVHD. One patient was lost to follow up.

### Induction Treatment

Ninety-seven patients had data regarding the first course of induction therapy. In this group of patients, 61.9% (60/97) received VDCP-based regimens, 17.5% (17/97) received hyper-CVAD A, 5.2% (5/97) received VDCP-based combined with chidamide regimens, 6.2% (6/97) received DAC combined with AAG priming regimen, and 9.3% (9/97) received other induction regimens. Complete remission (CR) was obtained in 74.2% (72/97) of patients after induction therapy, while CR after the first induction therapy was only 44.3% (43/97). A total of 3.1% (3/97) of patients died during the first induction therapy. Besides, in this study, we compared the differences in response among four different induction chemotherapy regimens (VDCP-based, Hyper-CVAD-A, VDCP-based combined with chidamide and DAC combined with AAG priming regimen) (**Supplementary Table 2**). Sixty patients received VDCP-based chemotherapy, including 30 patients with CR, 30 patients with NR. The CR rate was 50% (30/60). Seventeen patients received the hyper-CVAD A regimen, 4 patients received CR, 13 patients received NR, with a CR rate of 24% (4/17). Five patients received VDCP-based combined with chidamide regimen chemotherapy, 3 patients achieved CR, and 2 patients achieved NR, with a CR rate of 60% (3/5). Six patients received DAC combined with AAG priming regimen, 3 patients received CR, 3 patients received NR, with a CR rate of 50% (3/6). Among these patients, a pairwise comparison was made between the four groups. After adjustment by Bonferroni, there was no significant difference between the other groups (P > 0.125).

### Decitabine Combined With AAG Priming Regimen

A total of 12 patients received a decitabine combined with AAG priming regimen in this study, including 6 who were treatment-naïve and 6 who were relapsed/refractory. Among the 6 relapsed/refractory patients, 5 patients were primary refractory, and 1 patient relapsed after transplantation. After one course of reinduction, 3 (3/6) patients achieved CR, and 3 (3/6) patients remained NR. At subsequent follow-up, all 3 patients who received CR experienced recurrence, 1 relapsed during chemotherapy and 2 relapsed after allo-SCT. Moreover, 3 died as a result of disease progression, and 2 died from severe infection during the follow-up. The median survival of the 6 patients was 13.5 months (6–36 months), and only 1 patient remained in remission within the follow-up period.

### Allogeneic Hematopoietic Stem Cell Transplantation

**Figure 4:**
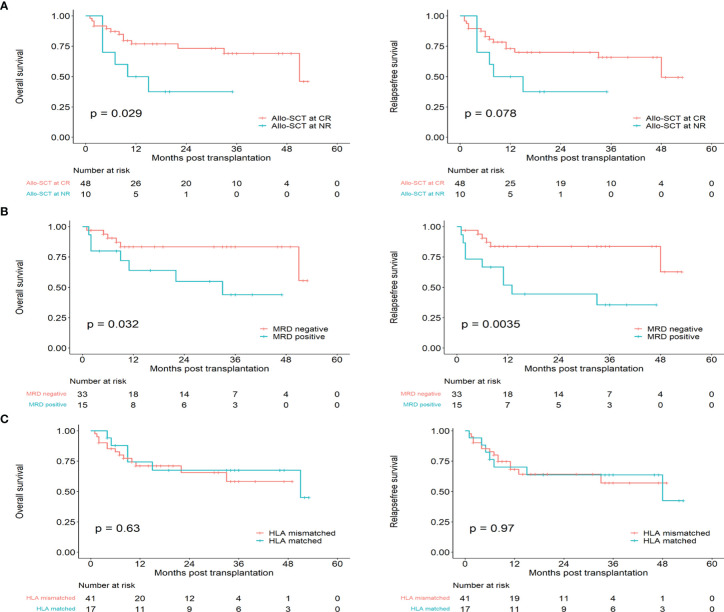
The prognosis of Early T-cell precursor leukemia/lymphoma with transplantation. **(A)** Overall survival and relapse free survival for allo-SCT at CR and allo-SCT at NR. **(B)** Overall survival and relapse free survival for patients with CR in MRD positive and MRD negative. **(C)** Overall survival and relapse free survival for HLA-matched and HLA-mismatched.

A total of 58 patients received allo-HSCT, including 46 CR1 patients, 2 CR2 patients and 10 NR patients. The median interval from CR to transplantation was 3.5 (range, 1-12) months. A total of 13.0% (6/46) of the patients experienced relapse after allo-HSCT at CR1, while 50% (1/2) of the patients experienced relapse after allo-HSCT at CR2. Patients who experienced transplantation in CR had better OS than transplantation in NR (P=0.029, HR: 0.3625, 95%CI 0.1005 to 1.308), while the RFS was no statistical significance. (P=0.078, HR:0.4431, 95%CI 0.1340 to 1.465) (**Figure 4A**). There were 33 MRD negative and 15 MRD positive patients in the 48 patients who transplanted in CR, and patients who were MRD negative at transplantation had better OS and RFS than those who were MRD positive (OS: P= 0.032, HR: 0.3306, 95%CI 0.09829 to 1.112; RFS: P=0.0035, HR: 0.2492, 95%CI 0.07993 to 0.7770) (**Figure 4B**). Additionally, 17 patients had HLA-matched donors, and 41 patients had HLA-mismatched donors. There was no statistical significance in the OS and RFS of HLA-matched and HLA-mismatched patients (OS: P=0.629, HR: 0.7996, 95%CI 0.3144 to 2.034, RFS: P=0.972, HR: 0.9844, 95%CI 0.3982 to 2.433) (**Figure 4C**). Forty patients received a BU/CY-based conditioning regimen, 18 patients received a TBI/CY-based conditioning regimen, and these two cohorts had no difference in OS and RFS (OS: P=0.570, HR:1.340, 95%CI 0.5125 to 3.503, P=0.770, HR:1.150, 95%CI 0.4577 to 2.887). Ten patients received salvage hematopoietic stem cell transplantation, and all received CR after transplantation. However, 3 patients died of recurrence, 1 patient died of GVHD, 2 patients died of severe infection, and only 2 patients were still in relapse-free survival. The median times for neutrophil engraftment and platelet recovery were 11 days (range, 7–28 days) and 14 days (range, 7–25 days), respectively. Two patients died of serious infection before reaching platelet recovery. The cumulative incidence of II-IV aGVHD was 12% (7/58). Notably, we observed that patients with MRD negative after 1 course of consolidation therapy had better OS and RFS (OS: P=0.014, HR: 0.1180, 95%CI 0.03414 to 0.4078; RFS: P=0.022, HR: 0.2058, 95%CI 0.06637 to 0.6382) (**Supplementary Table 3**). The main transplantation characteristics of the patients are listed in **Supplementary Table 4**. The comparison clinical data of allo-SCT and chemo alone are listed in **Supplementary Table 5**.

### Univariate and Multivariable Analyses

Prognostic factors affecting OS in 97 patients with ETP-ALL/LBL were analyzed by univariate Kaplan–Meier analysis and a Cox multivariate model analysis, the results are shown in **Table 2**. Age, gender, white blood counts, hemoglobin counts, platelet counts, LDH concentration, BM blasts, cytogenetics, the number of mutations, NOTCH1 mutation, presence of mediastinal involvement at diagnosis, disease status after the first course induction and allo-SCT were included in univariate and multivariable analyses. Age >39 years old, CR after the first course of induction therapy and allo-SCT were prognostic factors affecting OS in patients with ETP-ALL/LBL. A multivariate analysis showed that whether CR was received after the first course of induction (P < 0.001, HR: 0.24, 95% CI 0.12 to 0.46) and whether allo-SCT was performed (P < 0.001, HR: 0.25, 95% CI 0.13 to 0.48) were independent prognostic factors for ETP-ALL/LBL.

**Table 2:** Univariate and multivariate Analyses of overall survival (OS) for early T-cell precursor acute lymphoblastic leukemia/lymphoma (ETP-ALL/LBL).

Variables	Univariate	Multivariate	P value
	P value	HR (95% CI)	
Gender (male or female)	0.409	-	-
Age(years) (>39 or ≤39)	**0.038**	1.47 (0.69-3.11)	0.314
WBC(10^9/L) (≥100 or <100)	0.797	-	-
Hemoglobin (g/L) (≥100 or <100)	0.496	-	-
Platelet(10^9/L) (≥35 or <35)	0.545	-	-
LDH(U/L) (≥245 or <245)	0.971	-	-
BM blasts(%) (≥60 or <60)	0.414	-	-
Complex karyotype (Yes or No)	0.461	-	-
NOTCH1 mutation (Yes or No)	0.299	-	-
Number of mutations (≥5 or <5)	0.400	-	-
Mediastinal involvement (Yes or No)	0.200	-	-
CR after the first induction (Yes or No)	**<0.001**	0.24 (0.12-0.46)	**<0.001**
Allo-SCT (Yes or No)	**<0.001**	0.25 (0.13-0.48)	**<0.001**

WBC, white blood cell; LDH, lactate dehydrogenase; BM, bone marrow; CR, complete remission; Allo-SCT, allogeneic stem cell transplantation. The significant P-values are in bold.

Univariate and multivariate analyses were performed for the survival of ETP in 58 patients who received allo-SCT, including age, sex, MRD status at transplantation, transplantation type and conditioning regimen (**Table 3**). Patients with MRD negative at transplantation and transplantation at CR had better OS, and those with negative MRD at transplantation had better RFS. A multivariate analysis showed that MRD negative at transplantation was independent prognostic factors of OS and RFS for ETP-ALL/LBL with allo-SCT (OS: P= 0.042, HR: 3.30, 95% CI 1.05 to 10.40; RFS: P=0.006, HR: 4.57, 95%1.53 to 13.65).

**Table 3:** Univariate and multivariate analysis for OS and RFS in patients with transplantation.

Variables	Overall survival			Relapse free survival		
	Univariate	Multivariate		Univariate	Multivariate	
	P value	HR (95%CI)	P value	P value	HR (95%CI)	P value
Age(years) (>39 or ≤39)	0.259	-	-	0.212	-	-
Gender (male or female)	0.666	-	-	0.774	-	-
MRD status at transplantation (Negative or positive)	**0.006**	3.30 (1.05-10.40)	**0.042**	**0.001**	4.57 (1.53-13.65)	**0.006**
CR status at transplantation (CR or NR)	**0.029**	1.48 (0.49-4.47)	0.485	0.078	1.03 (0.36-2.91)	0.957
Transplantation type (HLA-matched or HLA-mismatched)	0.629	-	-	0.972	-	-
BU/CY regime	0.570	-	-	0.770	-	-

MRD, minor residual disease; CR, complete remission; NR, no response. The significant P-values are in bold.

## Discussion

To date, few studies involving multicenter cohorts of ETP-ALL/LBL have been reported, and this study is one of the largest cohorts of ETP type. In this study, we summarized the clinical characteristics of 103 patients with ETP-ALL/LBL, compared the efficacy of different induction regimens and hematopoietic stem cell transplantation, and explored the potential efficacy of treatment regimens for relapsed refractory ETP-ALL/LBL.

The patients’ characteristics in our study, such as the WBC counts, age of onset, sex, and initial response of induction, were similar to those in some previous studies (3, 16). AYA tended to have more splenomegaly and mediastinal masses in clinical manifestations than older adults. Next-generation sequencing revealed that the pediatric cohort presented a frequent profile of mutations with factors in regulating cytokine receptor and RAS signaling, hematopoietic development, and histone modification (6), which was similar to our results. In our study, the most frequently mutated gene was NOTCH1 (35.1%). However, the frequencies of NOTCH1 mutations in the ETP type vary worldwide (4, 17). In addition, Noronha et al. (17) found that a NOTCH1 mutation was associated with significantly better OS in their cohort, which was not consistent with our AYA and older adult cohort. We also found that patients had more frequent mutations in DNA methylation factor genes and myeloid neoplasms than the pediatric cohort. These differences might reflect age-related mutations, race distinctions and/or biological differences in hematopoietic progenitor cells during carcinogenic transformation in adults. However, Yang et al. (18) reported that patients with the SET-CAN fusion gene, which still has a limited understanding, had a poor prognosis. This finding was not observed in our study, in which the number of cases and severity of chemotherapy-related complications may influence the difference.

In this study, the CR rate was 74.2%, higher than the remission rate of another center in China (64.2%), but lower than the studies reported by Bond et al. (87.2%) and Morita et al. (83%) (5, 16, 19). We found that some patients in studies with higher CR rates received induction therapy containing nelarabine, which might account for the difference. To date, the survival outcome of ETP-ALL is still controversial. Some previous studies believed that ETP-ALL was a high-risk T-ALL with worse prognosis than non-ETP-ALL (1, 3), while some studies believed that the outcome of ETP-ALL was not significantly different from other T-ALL types (5, 9, 16). An important difference among different studies was the rate of allo-SCT. In our research, the 2-year OS rate was 54%, and the 2-year RFS rate was 57.7%, better than the results of the First Affiliated Hospital of Zhejiang University, another center in China (2-year OS: 40.7%, 2-year RFS: 47.2%) (5). The differences may be related to the percentage of allo-SCT, the age of patients and the effective induction regimen. In our study, more than half of the patients received allogeneic hematopoietic stem cell transplantation, and 81.4% of patients were younger than 39 years old.

ETP-ALL/LBL has a higher rate of remission failure and subsequent relapse than typical T- ALL, and the prognosis of R/R ETP-ALL/LBL was worse. In a multicenter study, 17 R/R T-LBL/ALL patients receiving chidamide combined with chemotherapy as salvage therapy reported a better response than those receiving chemotherapy alone (71% vs. 42%, P=0.037) after 1 cycle of induction (20). Yang et al. reported an ETP-ALL/LBL patient who showed primary resistance to lymphoid- and myeloid-directed induction therapy and finally achieved CR with a DAC-containing G-CSF priming regimen (21). In addition, Zhang et al. reported that 1 patient with R/R ETP-ALL was salvaged by the venetoclax plus HAG regimen (22). A DAC combined with AAG priming regimen demonstrated clinical antineoplastic activity in R/R ETP-ALL (CR: 50%) in our research. Patients who achieved CR after DAC combined with AAG priming regimen reinduction showed more myeloid gene mutations, such as DNMT3A, WT1, SETD2, KRAS, and U2AF1, than patients who achieved NR, further indicating that myeloid-based therapy might be suitable for the ETP subtype. A previous study has already demonstrated that decitabine can enhance the chemosensitivity of ETP cell lines (23). Further research is needed to distinguish effective methods. Currently, there is no standard regimen for relapsed/refractory ETP-ALL/LBL. A phase I/II study of ruxolitinib in combination with L-asparaginase, vincristine, and prednisone in adult patients with R/R ETP-ALL by Sichuan University is underway (NCT03613428).

Allo-SCT is an effective treatment that can cure hematological malignancies. In the GRAALL studies, allo-SCT in CR1 was recommended for patients with high-risk features, including corticosteroid resistance and early bone marrow chemotherapy resistance, and the 5-year OS of ETP was not inferior to that of the non–ETP-ALL group (59.6% vs. 66.5%, P=0.33) (16). Brammer et al. found that ETP patients transplanted in CR1 had an OS of 47%, comparable to other T-ALL disease subtypes, suggesting that allo-SCT can overcome the poor prognosis associated with ETP (9). Similarly, our results also showed that allo-SCT in CR can significantly improve the prognosis of ETP, especially in MRD negative patients. There was no significant difference in OS and RFS between HLA-matched and HLA-mismatched patients. However, salvage allo-SCT transplantation cannot maintain long-term survival. Exploring effective salvage treatment to improve the depth of remission and benefits from allo-SCT would be worthwhile. Chemotherapy combined with novel therapies, such as TKIs, JAK2 inhibitors, CD38 monoclonal antibodies, chimeric antigen receptor T cells targeting CD7 and Bcl-2 inhibitors, may help to achieve lower MRD levels and will hopefully lead to improved outcomes (24–28). Therefore, allo-HSCT should be performed as soon as possible for patients after achieving MRD negative results, and HLA-mismatched transplantation is also a worthwhile choice for patients without sibling donors and HLA-matched unrelated donors.

Although this was the largest cohort of ETP types, our study has several limitations. It is a multicenter retrospective study in which baseline characteristics were not not available for all patients and has a high rate of loss to follow-up, which may cause deviation in the prognostic analysis. To date, an open-label, one-arm, multisite trial to evaluate the safety and effect of chidamide for adult ETP-ALL/LBL is ongoing by Nanfang Hospital of Southern Medical University (NCT03553238). A clinical trial of decitabine combined with the HAAG regimen in newly diagnosed ETP-ALL/LBL by our center is also underway (NCT04446130).

In summary, ETP-ALL/LBL has a higher rate of remission failure and poor long-term prognosis, and there is no standard regimen for relapsed/refractory ETP. Effective induction chemotherapy regimens and targeted therapies need to be explored. Allo-SCT can abrogate the unfavorable impact of ETP type, particularly when performed in MRD negative. In addition, the presence of MRD at transplantation was a clear prognostic marker for increased disease relapse. Ultimately, a large registry analysis will be needed to validate these findings.

## Data Availability Statement

The raw data supporting the conclusions of this article will be made available by the authors, without undue reservation.

## Ethics Statement

The studies involving human participants were reviewed and approved by The First Affiliated Hospital of Soochow University. Written informed consent to participate in this study was provided by the participants’ legal guardian/next of kin. Written informed consent was obtained from the individual(s), and minor(s)’ legal guardian/next of kin, for the publication of any potentially identifiable images or data included in this article.

## Author Contributions

YX, HZ, LD and DW contributed to the conception of the study and the manuscript’s revision. JX, ZC, HW, XL, YL, YW, PX and LW collected data for the study. JX, ZC, HW, XL, BZ and YL participated in the analysis and interpretation of the data. JX, ZC, and HW participated in writing the manuscript. All authors reviewed the manuscript, approved the final version and support this publication.

## Funding

This work was supported in part by grants from the National Natural Science Foundation of China (81730003, 81870120, 82070187), the Social Development Project of Jiangsu Province (BE2019655), the Jiangsu Province Key R&D Program (BE2019798), the Priority Academic Program Development of Jiangsu Higher Education Institutions (PAPD), and the National Key Research and Development Program (2017ZX09304021, 2017YFA0104500, 2019YFC0840604).

## Conflict of Interest

The authors declare that the research was conducted in the absence of any commercial or financial relationships that could be construed as a potential conflict of interest.

## Publisher’s Note

All claims expressed in this article are solely those of the authors and do not necessarily represent those of their affiliated organizations, or those of the publisher, the editors and the reviewers. Any product that may be evaluated in this article, or claim that may be made by its manufacturer, is not guaranteed or endorsed by the publisher.
