# Supplementary material for: The Clinical Characteristics and Prognosis of AYA and Older Adult ETP-ALL/LBL: A Real-World Multicenter Study in China
Source: Front Oncol. 2022 Jun 6;12:846573. doi: 10.3389/fonc.2022.846573 (PMC9207171; doi:10.3389/fonc.2022.846573)
Supplement: Supplementary file 1 [file DataSheet_1.pdf]

Supplementary table 1 | Comparison of the characteristics of patients between AYA and older adult.

Variables	>39 years (n= 18)	≤39 years (n= 79)	P value
Sex			0.298
Male	11	58	
Female	7	21	
WBC × 10^9 /L	6.325(0.23-643)	9.09(0.22-258.52)	0.198
⩾100 × 109 /L	1	12	0.451
Hemoglobin, g/L	101(51-125)	103.5(41-172)	0.062
Platelet, 10^9 /L	104(17-413)	109(10-302)	0.063
LDH, U/L			
> 245, U/L	8	31	0.684
BM blasts (%)	80(7-97)	80(4.5-99)	0.100
Complex karyotype	2	14	0.728
Mediastinal mass involvement	1	23	**0.038**
Spleen enlargement involvement	3	40	**0.009**
Peripheral lymph node enlargement involvement	7	39	0.422
CR after the first induction	10	33	0.288

LDH, lactate dehydrogenase; BM, bone marrow; CR, complete remission.

Supplementary table 2 | The response of early T-cell precursor acute lymphoblastic leukemia / lymphoma (ETP-ALL/LBL) after induction chemotherapy

Induction therapy regime	N	CR rate	MRD status in CR		
			MRD negative	MRD positive	MRD NA
VDCP-based regime	60	50%	9(30%)	18(60%)	3(10%)
Augmented MDACC hyper-CVAD A protocol	17	24%	0(0%)	3(75%)	1(25%)
VDCP-based regime combined with chidamide	5	60%	2(67%)	1(33%)	0(0%)
Decitabine-containing G-CSF priming regimen	6	50%	2(67%)	1(33%)	0(0%)
Others	9	33.3%	0(0%)	2(67%)	1(33%)

CR, complete remission; MRD, minor residual disease; NA: unavailable.

Supplementary table 3 | MRD Dynamic Monitoring of ETP-ALL/LBL with allogeneic hematopoietic stem cell transplantation (allo-HSCT)

Time Frame	MRD positive (n)	MRD negative (n)	P(OS)	P(RFS)
MRD after induction	36	15	0.090	0.164
MRD after one course of consolidation	22	21	**0.014**	**0.022**
MRD at transplantation	15	33	**0.032**	**0.004**

MRD, minor residual disease; OS, overall survival; RFS, relapse-free survival.

Supplementary table 4 | The characteristics of early T-cell precursor acute lymphoblastic leukemia / lymphoma (ETP-ALL/LBL) with allogeneic hematopoietic stem cell transplantation (allo-HSCT)

**Transplant characteristics (n= 58)**	
**Characteristic**	**n (%)**
Disease status at transplant	
CR1	46(78%)
CR2	2(5%)
NR	10(17%)
MRD statue at transplantation	
MRD ≥ 10^-4	30(53%)
MRD < 10^-4	28(47%)
Stem cell source	
Peripheral blood	34(59%)
Bone marrow	2(3%)
P+B	21 (36%)
Unknown	1(1%)
Transplantation type	
HLA-matched	17(31%)
HLA-mismatched	41(69%)
Conditioning regimens	
BU/CY-based	40(69%)
TBI/CY-based	18(31%)
NC （10^8/kg）	9.55(3.18-25.65)
CD34+ (10^6/kg)	4.999(0.34-20.96)
aGVHD (II-IV)	7(12%)

CR1, complete remission for the first time; CR2, complete remission for the second time; NR, no response; MRD, minor residual disease; NC: nucleated cells; aGVHD, acute graft-versus-host disease.

Supplementary table 5 | Comparison of the characteristics of patients with allo-hematopoietic stem cell transplantation (allo-HSCT) or chemotherapy alone.

**Variables**	**Allo-HSCT (n = 58)**	**Chemo alone (n = 39)**	**P value**
Sex			0.651
Male	40(69.0%)	29(74.4%)	
Female	18(31.0%)	10(25.5%)	
Age, years			
> 39 years	4(6.9%)	14(35.9%)	**<0.001**
White blood cell, 10^9/L	9.715(0.22-258.52)	5.81(0.23-643)	0.520
≥100 × 109 /L	8(14%)	5(12.8%)	0.890
Hemoglobin, g/L	113(41-172)	88(51-145)	**0.042**
Platelet, 10^9 /L	109.5(12-273)	112(10-413)	0.510
LDH, U/L			
>245, U/L	20(34.5%)	19(48.7%)	0.206
BM blasts (%)	80(4.5-99)	76.5(5-99)	0.569
Induction therapy			0.519
VDCP-based regimens	34(58.6%)	26(66.7%)	
Hyper CVAD-A	13(22.4%)	4(10.3%)	
VDCP-based+ Chidamide	3(5.2%)	2(5.1%)	
DAC+G-CSF priming regimen	4(6.9%)	2(5.1%)	
Others	4(6.9%)	5(12.8%)	

LDH, lactate dehydrogenase; BM, bone marrow.
